# A Retrospective Review of Gynaecological and Social Outcomes for Teenage Pregnancies from 2020 to 2022 in Stoke-on-Trent

**DOI:** 10.3390/jcm14165745

**Published:** 2025-08-14

**Authors:** Maria van Veelen, Lauren Franklin, Aisling McCann, Fidelma O’Mahony

**Affiliations:** 1 Department of Obstetrics and Gynaecology, Royal Stoke University Hospital, University Hospitals of North Midlands NHS Trust, Stoke-on-Trent ST4 6QG, UK; 2 Faculty of Medicine and Health Sciences, Keele University, Keele ST5 5BG, UK

**Keywords:** adolescent, pregnancy, teenage, conception, obstetrics, complications

## Abstract

**Background/Objectives**: Adolescent pregnancies are associated with negative outcomes of health and social and economic consequences for both the mother and child. The aim of this audit was to determine the incidence of gynaecological complications and adverse social determinants of health affecting pregnant women less than 20 years old at the time of delivery in Stoke-on-Trent, with the goal of improving local and national trust guidelines. **Methods**: A retrospective case note review was conducted using electronic databases. Subjects had to be under the age of 20 years old at the time of delivery at our local tertiary hospital from January 2020 to December 2022. **Results**: Four hundred and seventy-three women met the inclusion criteria. The median age was 18 years old (range: 13–19 years). Most women delivered at term (mean 38^+3^), were primigravida (76%), and underwent spontaneous delivery (43%). Both our induction rate of 28.2% and caesarean section rate of 18.4% were below the national averages. Complications of post-partum haemorrhage and low birth weights exceeded the national averages, with third- to fourth-degree perineal tears just below the national incidence rate of 2.9%. Negative social determinants of health included smoking, mental illness, and low breastfeeding rates. Our mean 3-year breastfeeding rate was 24.3%. **Conclusions**: This single-centre audit at a large tertiary hospital has demonstrated that women under the age of 20 years old in socially deprived areas of the UK are more likely to experience negative gynaecological and social outcomes from their pregnancies compared to areas of low deprivation within the UK.

## 1. Introduction

Adolescent pregnancies are associated with negative outcomes of health and social and economic consequences for both the mother and child, including higher risks of eclampsia, systemic infections, low birth weight and preterm birth [1,2,3,4,5,6]. The global adolescent birth rate (ABR) has been decreasing over the past two decades, but there remains great variability across different continents, and even among regions within developed countries [1]. Moreover, despite the reductions made in global ABR, teenage pregnancy conception rates remain high [1].

Many studies have demonstrated that women under the age of 20 with less education and/or from lower socioeconomic backgrounds are more likely to experience adolescent pregnancy, with worse gynaecological and social outcomes for teenagers from low-income areas [1,2,4,6,7]. Furthermore, those living in areas with high deprivation are more likely to have difficulties accessing contraceptive services and experience child sexual abuse, both leading to unintended pregnancies [1,6,7]. They have also been found to be at a greater risk of suffering mental health problems, committing suicide, smoking and using illicit drugs during their pregnancies [6,7,8]. Worldwide, delivery complications from teenage mothers undergoing childbirth remains one of the leading causes of death amongst adolescents [1,6].

Political policies to reduce teenage pregnancies have shown to be more effective in areas of low deprivation compared to regions of high deprivation [1,6,7,8,9]. The government of the United Kingdom (UK) introduced their Teenage Pregnancy Strategy in 1999 to tackle the burdens of adolescent pregnancies [6,9,10]. The aim of the programme was to reduce the pregnancy rates in women under the age of 18 over the next 10 years by 50%, while also boosting National Health Service (NHS) support to teenage mothers [6,9,10]. The programme was hailed as a success given its 51% reduction in the under-18 conception rate, especially compared to European counterparts that only experienced a 28% reduction rate during the same period, but when the programme came to an end in 2010, critics have noted some key factors which may have overestimated its success [6].

The UK Teenage Pregnancy Strategy was extravagantly costly, with over £60 million Great British Pounds (GBP) spent within the first three years of the programme [6]. The ten-year focus of improving local trust guidelines, health and education programmes, and easily accessible contraceptive services did not continue after the policy’s expiration [6]. Critics argue that a major focus of the strategy was teenager ignorance towards pregnancy; however, improving sex education in communities was less effective than challenging the underlying societal influences which maintain inequalities within areas of high deprivation [6]. These claims are supported by ongoing evidence that adolescents in England are still experiencing high teenage birth rates, higher risks of unintended pregnancies, child poverty, and inequalities exist between local authorities, making birth outcomes for British adolescents and their children inordinately poor [8,9,10,11].

Although the conception rate of teenage pregnancies in the UK has been decreasing since 2007, reducing by more than half since 2011, numbers were on the rise again in 2021 [5,6,7,11]. Despite reductions in key areas, such as London, the prevalence of teenage pregnancy in the UK remains high in socially deprived areas and the UK still has one of the highest numbers of teenage pregnancies per year in Western Europe [5,7]. The Northeast of England has had the highest teenage conception rates of all English regions since 2003, averaging 19.8 conceptions per 1000 women in 2021 [7,11]. Although the West Midlands of England averaged a teenage conception rate of 15 per 1000 women in 2021, Stoke-on-Trent’s rate was 24.4 conceptions per 1000 women [11].

## 2. Materials and Methods

A single-centre retrospective audit was conducted between January 2020 and December 2022. The primary aim was to measure the gynaecological and social outcomes of teenage pregnancies to improve local and national trust guidelines. All patients under the age of 20 years at time of delivery at Royal Stoke University Hospital from January 2020 to December 2022 were included. Initial coding was provided by the Obstetrics & Gynaecology Directorate of Clinical Audits of the University Hospitals of North Midlands and data collection was accessed using electronic medical records.

The World Health Organization (WHO) and the Royal College of Obstetricians and Gynaecologists (RCOG) define teenage pregnancy conception rates for any woman as under the age of 20 years [1,12], but the UK Office of National Statistics (OFNS) and Public Health England (PHE) define their teenage conception rates for women as under the age of 18 years [9,11]. For the purposes of this audit, our inclusion criteria specified teenage women as under the age of 20 years. National incidence data from the OFNS, RCOG, and the NHS was comparatively used to evaluate the complication rates and social outcomes in our population. Birth categories were defined by the WHO criteria of extremely preterm (<28 weeks), very preterm (28 to <32 weeks), moderate to late preterm (32 to 36^+6^ weeks), term (37^+0^ to 41^+6^ weeks) and post-term (≥42^+0^ weeks) [13,14].

## 3. Results

Four hundred and seventy-three women met inclusion criteria. The final retrospective review involved 175 patients in 2020, 137 in 2021, and 161 in 2022. Most women fell into the 18-year-old (33%) and 19-year-old (28%) age brackets, but there was one 13-year-old and two 14-year-olds in 2022 [Table 1]. The other age brackets, 15-year-olds (5.3%), 16-year-olds (9%) and 17-year-olds (24%), were evenly distributed over the three-year period [Table 1]. The mean gestation at delivery was 38^+3^. Those in the extremely preterm category accounted for 3.4% of all births, very preterm was 0.6%, moderate to late preterm was 6.4%, term was 87.4% and post-term was 2.2% of all deliveries. Most women were primigravida (361; 76%). There were 89 women as gravida (G) 2 (19%), 21 as G3 (4.4%), with one G5 and one G6 in 2022.

### 3.1. Modes of Delivery

Most women (43%) experienced spontaneous vaginal (SV) delivery [Table 2]. Of the 135 women who required induction of labour (IOL), the main reasons for induction included failure to progress/delay in second stage, foetal growth restriction (FGR), reduced foetal movement (RFM) and reduced growth velocity (RGV). When forceps or a vacuum device were required in 48 deliveries, the main indications were cardiotocography (CTG) concerns suggesting foetal compromise, prolonged latent phase, pre-eclampsia and shoulder dystocia.

Emergency caesarean sections (EMCS) were necessary for 75 women due to failed inductions for prolonged first or second stage, RFM, preterm premature rupture of membranes (PPROM), placental abruption and obstetric cholestasis [Table 2]. Elective CS was scheduled for 12 women due to previous EMCS, large for gestational age (LGA), abnormal middle cerebral artery (MCA) Doppler, patient preference and breech.

### 3.2. Delivery Complications

Just under a third (139; 29.4%) of our population experienced postpartum haemorrhage (PPH) [Table 3]. A much lower proportion suffered with pre-eclampsia (26; 5.5%) and eclampsia (2; 0.4%) [Table 3]. On the contrary, low birth weights had a higher incidence (11%) from 54 live births [Table 3]. Perineal tears had an overall incidence rate of 39.6% (189) with 12 women suffering from third-degree tears (2.5%) and one woman with a fourth-degree tear (0.2%) [Table 3].

### 3.3. Neonatal Deaths and Stillbirths

From the 175 babies delivered in 2020, there were two early neonatal deaths. One was caused by spontaneous ROM (SROM) at 23^+0^ weeks. The other baby was born with congenital diaphragmatic hernia (CDH) with a deviated heart at 21^+3^and passed away within 90 min.

From the 139 babies delivered in 2021, there was one early neonatal death, one miscarriage, one stillbirth, and three medical terminations of pregnancy (MTOP) for foetal abnormalities. The early neonatal death was from a baby born at 22^+5^ who later died in the Neonatal Unit (NNU). Histology later indicated separation of the placenta from the uterine wall suggesting infection. The unexpected miscarriage was caused by chorioamnionitis at 15^+2^ from suspected maternal Rubella infection. The stillbirth occurred at 23^+4^ weeks after the mother self-discharged against medical advice (AMA) with raised blood pressure and subsequently returned after three seizures, confirming eclampsia. The first MTOP was for confirmed Cystic Fibrosis (CF) at 21^+0^. The second was for confirmed omphalocele at 16^+2^, but the third was an unlisted suspected anomaly at 17^+6^.

From the 163 babies delivered in 2022, there were two miscarriages and three antepartum stillbirths. The first miscarriage was a missed miscarriage from a set of twins at 13^+6^ whereas the second miscarriage had no known cause detailed in the notes at 16^+2^. Two stillbirths required IOL for intrauterine death, one at 40^+0^ and the other at 28^+4^. The third stillbirth occurred after the mother had a suicide attempt with a paracetamol overdose at 20^+6^.

### 3.4. Social Aspects

A third of our women (148; 31.3%) suffered from mental health conditions and this was worsening to 40% (64/161) in 2022. Anxiety and depression were the most common psychiatric conditions, followed by personality disorders and post-traumatic stress disorder (PTSD) from sexual abuse at young ages. One out of every five of our women (101; 21.4%) required social services. Reasons for social work involvement included unsafe or temporary housing, domestic violence, learning disabilities, drug abuse, previously being in foster care, decision to place the baby in foster care, and/or risk of child exploitation.

Almost a third (130; 27.5%) of our population stated they were current smokers at their booking appointment, with another third (141; 29.8%) stating they previously smoked. Of those identified, 85 (18%) reported a diagnosis of asthma. Ninety-nine women (21%) stated they drank alcohol weekly prior to their pregnancy, with 69 (14.6%) reporting previous illegal substance abuse. Of those revealing active substance abuse during their pregnancies (5; 10.6%), there were two women in 2020 and three women in 2022. For 2020, one patient used cocaine and ecstasy in their first trimester prior to discovering she was pregnant, and the other patient was on methadone for previous addictions to benzodiazepines, cannabis, cocaine, heroin and ketamine. In 2022, all three patients actively smoked cannabis during their pregnancies. For sexually transmitted infections (STIs) reported at their initial booking appointment, there was one in 2020, two in 2021, and six in 2022. Of those, six had previously treated chlamydia, one had chlamydia and gonorrhoea at the age of 13, one had genital herpes simplex virus (HSV), and one was successfully treated for syphilis.

Our breastfeeding rates declined over the three-year period with an overall average of 24.3% [Table 4]. Although we confirmed first feed with the initial health visitor check at 10–14 days as well as the six-week baby check, we were unable to confirm if breastfeeding continued after this time. If mothers provided breastmilk on the first feed, but then switched to formula immediately after this, they were only included in the formula group. After excluding neonatal deaths, 11 (2.3%) women’s feeding methods were found to be not documented in their medical records [Table 4].

## 4. Discussion

To our knowledge, this is the first published audit of local teenage pregnancy outcomes in the West Midlands of England. The results signify several key indicators. Although research has clearly demonstrated that pregnant women under the age of 20 are more at risk of delivering pre-term [1,3,7,12,13,14,15], our data suggests that most women in our population will make it to term if they attend their structured antenatal appointments. However, our combined pre-term rates surmounted to 10.4%. The national rate of preterm birth for all ages in England had increased to 7.9% in 2022, worse yet in Wales at 8.1%, and 8.4% in Scotland, with estimations suggesting they will continue to rise after 2025 [14]. The risk factors for preterm delivery have been deemed multifactorial, and those found in our population to be possible contributors are low maternal reproductive age, socioeconomic deprivation, pre-existing mental health conditions, and especially tobacco smoking [14]. Limitations of our data which also could have influenced preterm deliveries were the body mass index (BMI) values and ethnicity of the mothers.

The incidence rate of SV deliveries in our population at 43% was equivalent to all age groups in England for 2020 to 2022 at 47% [16]. Our induction rate of 28.2% was below the national average of 33% and our rate of CS (18.4%) fell just below the national average of 20% [16]. However, our complications compared to national standards were more varied.

PPH is defined as ≥ 500 mL of blood loss within 24 h after the birth of a baby and is the most frequently occurring form of obstetric haemorrhage [17]. Although our incidence rates of PPH were reducing over the three-year period, they remained above the national average of 25% [16]. Conversely, our results for pre-eclampsia and eclampsia were in-line with national incidence rates [13]. Perineal tears (39.6%) were just above England’s averages, as perineal lacerations occurred at rates as high as 39% from 2021 to 2022, but our third- and fourth-degree tears were just below the national incidence rate of 2.9% [18].

Low birth weight is defined by the WHO as a live birth weighing less than 2500 g irrespective of their gestational age [16]. Although our incidence rate of low birth weights decreased by nearly half in 2022 to 7.5%, our three-year average of 11.4% was well above the national rate of 7% for neonates weighing below 2500 g [16]. Previous research measuring the confounding factors affecting adverse birth outcomes such as low birth weight in teenage pregnancy remains conflicting [3,7,11,12,14,15]. True measures of social deprivation and their contributions to low birth weight in teenage pregnancy have been disputed, with smoking being the greatest instigating factor associated with low birth weight in teenage deliveries [2,3,4,6,15].

Across the UK, neonatal deaths have been reducing since 2010, with the stillbirth rate at 4.0 per 1000 births and the neonatal death rate at 2.9 per 1000 births in 2022 [19]. However, there is a stark difference within areas of high and low deprivation. In 2022, when comparing the most deprived areas to the least deprived areas, stillbirth rates were 4.6 per 1000 total births compared to 2.61 per 1000 total births, and neonatal mortality rates for the most deprived areas were double that of the least deprived areas at 2.38 per 1000 live births compared to 1.18 per 1000 live births [19,20]. Our results of stillbirths and early neonatal deaths, especially in 2022, exceed the values for the most deprived areas in the UK and exemplify a growing concern for our population. The aetiology of these deaths is multifactorial, and although mental health and substance abuse existed in a few of our patients with early neonatal deaths, most causes were from congenital anomalies, with Rubella infection being one of the few conditions we could target in our female population through vaccination primary prevention prior to conception.

For all age groups within the UK, the rate of mothers actively smoking during their pregnancy was 9% in 2022, whereas for teenage mothers it was as high as 28% in England and Scotland [14]. Although our results are equivalent, it is unknown if the additional 141 women who stated they smoked prior to their initial booking appointment did in fact continue to smoke through their pregnancy. Additionally, smoking tobacco during adolescence has been linked to a fourfold increase in the likelihood of developing asthma, and smoking during pregnancy significantly increases the baby’s risk of developing asthma [14,15]. Nevertheless, the confounding factors of the 18% of our population with asthma are unknown, especially given that a study performed in 2023 determined Stoke-on-Trent to have the worst air pollution levels in all of the UK [21].

The most concerning findings of our audit were breastfeeding outcomes. National data for England states that 73.1% of teenage mothers under the age of 20 provide breastmilk on the first feed, but only 3% exclusively breastfed in 2022 [16]. However, 67% of the 12,095 women under the age of 20 who conceived in England in 2022 had values missing, so their breastfeeding status could not be commented on [16]. The stark difference in teenage mothers who are encouraged to breastfeed with the first feed but quickly change to formula (73% to 3%) was the reason we chose not to include exclusive first feed in our breastfeeding rates. We surmise that this could potentially indicate that teenage mothers are not educated in advance throughout their pregnancy of the pros and cons of breastfeeding, they may not feel confident enough to continue once it is experienced, and most importantly, there are too many social barriers keeping them from continuing to breastfeed, mostly the lack of safe privacy breastfeeding spaces in Stoke-on-Trent. More qualitative data is required for our population though as the biggest contributing factor found by our breastfeeding midwife team is that there are now generations of women in deprived areas who have exclusively formula-fed. Therefore, when a pregnant adolescent’s mother and grandmother did not breastfeed, this is a much greater influence on them than any professional medical advice given.

Although the outcomes of our audit demonstrated that our trust guidelines for teenage pregnancies were providing a structured and evidence-based antenatal care service to improve outcomes for our patients, primary prevention is missing both locally and nationally. Primary prevention, sex education, and access to contraceptive services are vital given that over half of teenage pregnancies are unintentional and end in abortion [1,10,22]. Adolescent pregnancy outcomes in areas of deprivation initially improved under the UK government Teenage Pregnancy Prevention Framework, but are now getting worse again, with Scotland’s teenage pregnancy rate being three times higher in areas of deprivation [6,7,8,11,14,20,22]. Moreover, when compared to other high-income English-speaking countries, many studies have refuted the claim that the Teenage Pregnancy Prevention Framework made an impact, and that changing societal influences of social deprivation would make a far greater impact than sex education and contraceptive services [6,14,20,22]. Whether numbers rising again in 2021 marks a key point of the UK government’s teenage pregnancy scheme coming to an end with cessation of funding, or this was an influence of societal change, is yet to be determined.

This study has design limitations as a single-centre service evaluation audit. Limitations include a lack of statistical analysis and external validity, the potential for systematic bias and that the results may not be generalisable to other trusts depending on their policies and practices. Further analyses are required for teenage pregnancies within the UK, including of the teenage mother’s contraceptive knowledge and their use at the time of conception and teenage views of breastfeeding and barriers to breastfeeding within areas of high deprivation in the UK.

## 5. Conclusions

In conclusion, this single-centre audit at a large tertiary hospital has demonstrated that women under the age of 20 years old in socially deprived areas of the UK are more likely to experience negative gynaecological and social outcomes from their pregnancies. The current national and local funding allocation to prevent teenage pregnancies requires updates regarding improved education and access to contraception services, as well as mental health services.

## Figures and Tables

**Table 1:** Age Distribution.

Age at Delivery (Years Old)	2020	2021	2022
19	42	41	49
	24%	30%	30.4%
18	60	44	52
	34%	32%	32.3%
17	52	33	29
	30%	24.1%	18%
16	11	13	19
	6.3%	9.5%	11.9%
15	10	6	9
	5.7%	4.4%	5.6%
14	0	0	2
			1.2%
13	0	0	1
			0.6%

**Table 2:** Modes of Delivery.

Modes of Delivery *	2020	2021	2022
Spontaneous Vaginal (SV)	70	66	68
	39.8%	48.2%	42.2%
Vaginal with Induction of Labour (IOL)	52	33	50
	29.5%	24.1%	31.1%
Instrumental Vaginal	23	12	13
	13.1%	8.8%	8.1%
Caesarean Section (CS)	31	26	30
	17.6%	18.9%	18.6%
a. Emergency	26	23	26
b. Elective	5	3	4

* 2020 included two sets of twins with 1 set delivered via SV and 1 set by EMCS; 2021 had two sets of twins delivered via EMCS; 2022 had two sets of twins with 1 set delivered via SV and 1 set by vaginal with IOL.

**Table 3:** Delivery Complications.

Complications	2020	2021	2022
Postpartum haemorrhage	53	41	45
	30.3%	29.9%	28%
Pre-eclampsia/Eclampsia	12/1	7/1	7/0
	6.9%/0.6%	5.1%/0.8%	4.3%/0%
Low birth weights	24	18	12
	13.6%	13%	7.5%
Perineal Tears	48.6%	37.2%	33%
a. First degree	7	10	13
b. Second degree	74	39	33
c. Third degree	4	2	6
d. Fourth degree	0	0	1

**Table 4:** Feeding Methods.

Feeding Methods	2020	2021	2022
Breastmilk	50	32	33
	28.6%	23.4%	20.5%
Formula	111	87	111
	63.4%	63.5%	68.9%
Mixed Breastmilk & Formula	9	7	9
	5.1%	5.1%	5.6%
Not documented/Not applicable *	5	11	8
	2.9%	8.0%	5.0%

* Not applicable related to neonatal deaths.

## Data Availability

The data presented in this study are available on request from the corresponding author so that the publication of such data does not compromise the anonymity of the participants or breach local data protection laws due to patient personal information.
